# Discovery and Validation of a Novel Metastasis-Related lncRNA Prognostic Signature for Colorectal Cancer

**DOI:** 10.3389/fgene.2022.704988

**Published:** 2022-05-19

**Authors:** Qiang Tang, Xin Hu, Qiong Guo, Yueyue Shi, Liming Liu, Guoguang Ying

**Affiliations:** ^1^ Laboratory of Cancer Cell Biology, National Clinical Research Center for Cancer, Key Laboratory of Cancer Immunology and Biotherapy, Tianjin’s Clinical Research Center for Cancer, Tianjin Medical University Cancer Institute and Hospital, Tianjin, China; ^2^ Department of Epidemiology and Biostatistics, National Clinical Research Center for Cancer, Key Laboratory of Cancer Prevention and Therapy of Tianjin, Tianjin’s Clinical Research Center for Cancer, Key Laboratory of Molecular Cancer Epidemiology, Tianjin Medical University Cancer Institute and Hospital, Tianjin, China

**Keywords:** colorectal cancer, metastasis, lncRNA, prognostic signatures, chemotherapy

## Abstract

**Background:** Cancer metastasis-related chemoresistance and tumour progression are the leading causes of death among CRC patients. Therefore, it is urgent to identify reliable novel biomarkers for predicting the metastasis of CRC.

**Methods:** The gene expression and corresponding clinical data of CRC patients were downloaded from The Cancer Genome Atlas (TCGA) and Gene Expression Omnibus (GEO) databases. Univariate and multivariate analyses were performed to identify prognostic metastasis-related lncRNAs. Nomograms were constructed, and the predictive accuracy of the nomogram model was assessed by ROC curve analysis. Then, the R package “pRRophetic” was used to predict chemotherapeutic response in CRC patients. In addition, the CIBERSORT database was introduced to evaluate tumour infiltrating immune cells between the high—and low-risk groups. The potential roles of SNHG7 and ZEB1-AS1 in CRC cell lines were further confirmed by *in vitro* experiments.

**Results:** An 8-lncRNA (LINC00261, RP1-170O19.17, CAPN10-AS1, SNHG7, ZEB1-AS1, U47924.27, NIFK-AS1, and LINC00925) signature was constructed for CRC prognosis prediction, which stratified patients into two risk groups. Kaplan-Meier analysis revealed that patients in the higher-risk group had a lower survival probability than those in the lower-risk group [*p* < 0.001 (TCGA); *P* = 0.044 (GSE39582); and *P* = 0.0078 (GSE29621)] The AUCs of 1-, 3-, and 5-year survival were 0.678, 0.669, and 0.72 in TCGA; 0.58, 0.55, and 0.56 in GSE39582; and 0.75, 0.54, and 0.56 in GSE29621, respectively. In addition, the risk score was an independent risk factor for CRC patients. Nomograms were constructed, and the predictive accuracy was assessed by ROC curve analysis. This signature could effectively predict the immune status and chemotherapy response in CRC patients. Moreover, SNHG7 and ZEB1-AS1 depletion significantly suppressed the colony formation, migration, and invasion of CRC cells *in vitro*.

**Conclusion:** We constructed a signature that could predict the metastasis of CRC and provide certain theoretical guidance for novel therapeutic approaches for CRC.

## Introduction

CRC is one of the most typical causes of cancer mortality and remains a challenging issue globally (Schmoll et al., 2012). Tumour metastasis is still the dominant cause of mortality among CRC patients (Wang et al., 2019). The survival rate of CRC is overtly relevant to clinical stage, and the 5-year survival rates of patients with no metastasis, local metastasis and distant metastasis are 90, 70 and 10%, respectively, (Ntavatzikos et al., 2019). Prognostic assessment and treatment decisions depend mainly on the pathological stage of the tumour, especially whether the tumour is accompanied by distant metastasis (Vetro et al., 2014). Therefore, reliable and robust new key molecules involved in the metastasis of CRC are urgently needed to improve personalized therapy for CRC patients.

LncRNAs comprise a large and diverse group of noncoding RNA transcripts longer than 200 nucleotides (Jarroux et al., 2017; Qian et al., 2020). Accumulating evidence shows that aberrant expression of lncRNAs is involved in the development and progression of tumours, mainly by epigenetic transcriptional modulation of coding genes or other lncRNAs (Yang et al., 2014; Bhan et al., 2017; Peng et al., 2017; Chan and Tay, 2018; Zhang and Tang, 2018). HOX antisense intergenic RNA (HOTAIR), one of the most representative lncRNAs, is strongly associated with multiple types of tumours (Qu et al., 2019; Rajagopal et al., 2020). Its high expression is associated with cancer metastasis in malignant tumours. Metastasis-associated lung adenocarcinoma transcript 1 (MALAT1) is also a widely studied lncRNA that participates in cancer progression and metastasis (Li Z.-X et al., 2018; Sun and Ma, 2019). The prognostic significance of MALAT1 has been revealed in cancers of the breast, lung, pancreas, and prostate. LncRNA HOXD cluster antisense RNA 1 (HOXD-AS1) has recently been discovered to be upregulated in many cancers, such as gastric cancer, hepatocellular carcinoma, and colorectal cancer (Li L et al., 2018; Xie et al., 2019). High expression levels of lncRNA HOXD-AS1 were associated with a high tumour node metastasis stage. LncRNA LINC02273 was recently identified as a metastasis-promoting gene and is significantly upregulated in metastatic lymph nodes (LNs) in breast cancer (Xiu et al., 2019). These studies demonstrate that lncRNAs may serve as novel biomarkers with diagnostic and prognostic value for cancer patients.

In the present study, we constructed an eight metastasis-associated lncRNA prognostic signature that may be a novel and independent prognostic factor of CRC patients based on TCGA dataset, and the predictive capacity was confirmed by the validation set (GSE39582, GSE29621). This signature could effectively predict the chemotherapy response in CRC patients.

## Materials and Methods

### Data Collection and Pre-Processing

**FIGURE 1:**
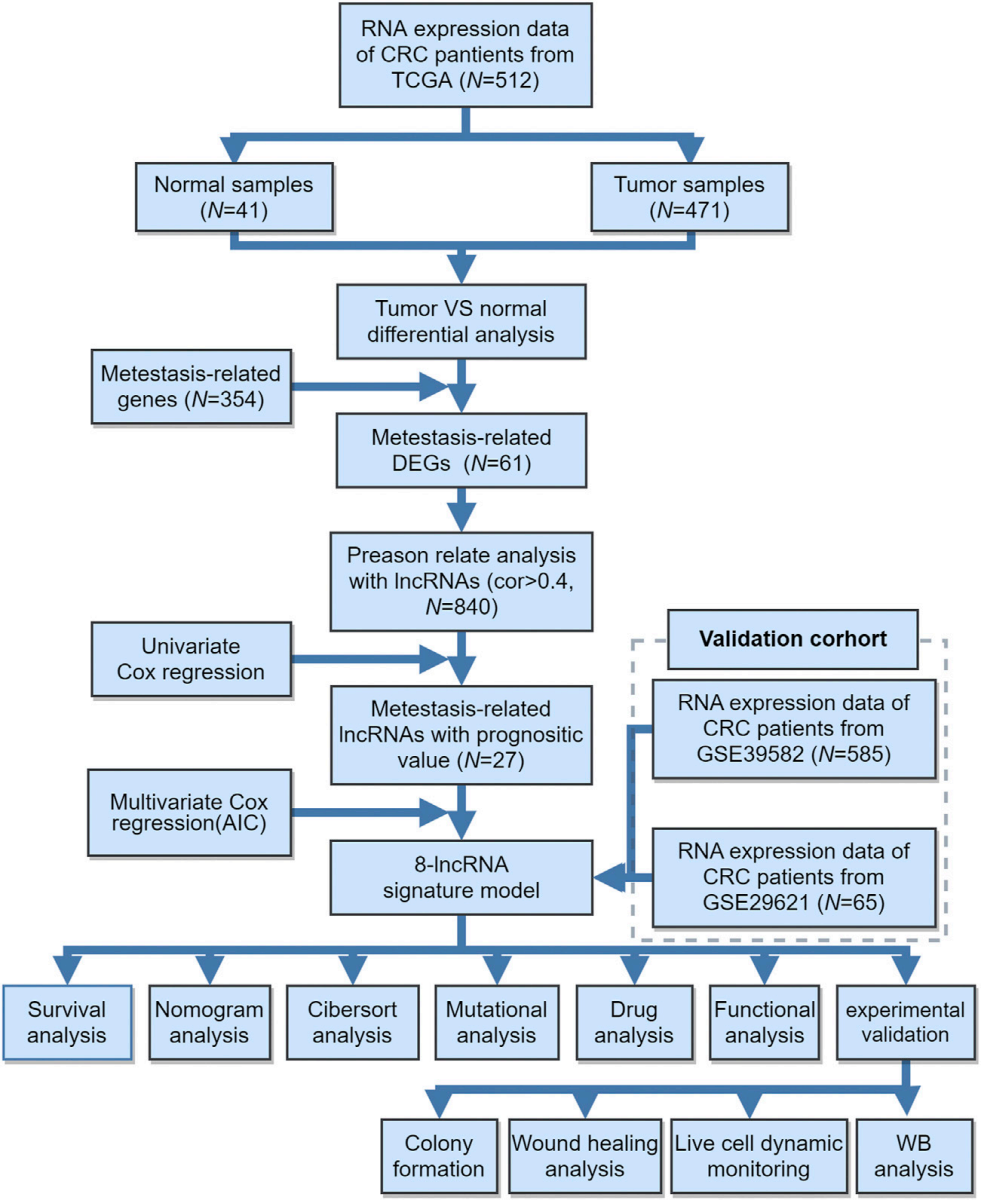
Overview of the study design and analysis of metastasis-related lncRNAs.

The workflow of this study is shown in Figure 1. The present study was based mainly on analysis of public gene expression data from TCGA and the GEO database. The fragments per kilobase of per million (FPKM) datasets of CRC RNA-seq and corresponding clinical information were downloaded from the UCSC Xena browser (http://xena.ucsc.edu/). Patients without corresponding survival information were removed from further evaluation. We downloaded the raw “CEL” files and performed background adjustment and quantile normalization by a robust multiarray averaging method with the affy package. Based on the lncRNA information in the GENECODE data resource V22 (https://www.gencodegenes.org/), lncRNA expression profiles were extracted from the RNA-seq cohort. In the present study, TCGA-COAD (TCGA-Colon Adenocarcinoma) datasets and GEO datasets (GSE39582, GSE29621) were used for training and validation sets, respectively. The differential expression of lncRNAs between CRC and normal tissues from the GEO database was downloaded from LncRNA Explore (https://lncar.renlab.org/explorer).

### Identification of Metastasis-Related lncRNAs in CRC

We identified the metastasis-related lncRNAs following three steps: First, 354 metastasis-related genes were obtained from the MSigDB Team (M9016 and M4100, respectively) (http://www.broad.mit.edu/gsea/msigdb/) (Provenzani et al., 2006). Second, we used the bioconductor “limma” package to calculate differentially expressed mRNAs based on RNA-seq data of TCGA normal samples and tumour samples. Differentially expressed mRNAs were determined using the cut-off thresholds of *p* < 0.05 and |log2-fold change| > 1. Third, the intersection of the above two genes were considered a metastasis-associated gene. Then, the Pearson correlation coefficients between the expression lncRNAs and the metastasis-related genes were calculated. LncRNAs with correlation coefficients >0.4 and *p* < 0.05 were identified as metastasis-related lncRNAs.

### Risk Assessment Model Construction and Prognostic Survival Analysis

We performed univariate Cox regression to calculate the prognostic metastasis-related lncRNAs. A *p* value less than 0.05 was considered significant. Metastasis-related lncRNAs were subjected to univariate Cox regression assessment to identify those linked to CRC overall survival (OS). Only lncRNAs with statistical significance (*p* < 0.05) were enrolled in multiple stepwise regression analysis. A risk assessment model for the patients was then developed using multivariate regression coefficients of lncRNA expression. Thus, we generated the risk score by combining the expression value of the included lncRNAs weighted by the linear regression model coefficients.

The risk scores of CRC patients were calculated using the risk assessment model. The patients were assigned to a high- and a low-risk group, respectively, based on the cut-off values calculated by the survminer package in R. The Kaplan-Meier method was used to assess the efficiency of OS in high- and low-risk patients. A log-rank test *p* < 0.05 was considered statistically significant. We also performed the same prognostic survival analysis in the validation cohort from GEO.

### Gene Sets Enrichment Analysis

We performed GO (gene ontology) enrichment analysis using the “clusterProfiler” R package to investigate the biological process differences between different risk groups. An adjusted *p* value less than 0.05 was considered statistically significant.

### Prediction of Chemotherapy Drug Response

The R package “pRRophetic” was used to predict chemotherapeutic response in CRC patients. The half-maximal inhibitory concentration (IC50) of the samples was calculated by ridge regression, and the prediction accuracy was assessed using 10-fold cross-validation based on the GDSC training set.

### Evaluation of Tumour-Infiltrating Immune Cells Between the High- and Low-Risk Groups

To evaluate the proportions of tumour-infiltrating immune cells in the tumour microenvironment (TME), the CIBERSORT database was introduced to quantify the relative infiltration of immune cells in the TME. CIBERSORT includes molecular characterizations of 22 tumour-infiltrating immune subsets in cancer. We obtained significantly different immune cell types between high- and low-risk patients.

### Cell Lines and Transfection

Cells were cultured in DMEM (Boster, China) with 10% FBS (HyClone, United States) in a humified atmosphere of 95% O_2_ and 5% CO_2_. Scrambled siRNAs were purchased from Shanghai GenePharma. Subsequently, cells were transfected with a Lipo3000 kit according to the manufacturer’s instructions.

### Western Blotting

Cells were incubated in ice-cold lysis buffer containing phosphatase inhibitor and protease inhibitor cocktail for 20 min. The bicinchoninic acid (BCA) assay was used to detect protein concentration. Equal amounts of protein samples were separated by 4–12% SDS/PAGE and transferred to PVDF membranes. The membranes were blocked with 5% nonfat dry milk for 1 h and incubated with the corresponding primary antibodies (GAPDH (1:1,000, Santa Cruz), vimentin (1:1,000, Santa Cruz), E-cadherin (1:1,000, Santa Cruz), N-cadherin (1:1,000, Santa Cruz), AKT/p-AKT (1:1,000 dilution, CST), ERK/p-ERK (1:1,000 dilution, CST), and PI3K/p-PI3K (1:1,000 dilution, CST) overnight at 4°C, followed by incubation with secondary antibodies for 1 h at room temperature. Immunoreactive protein levels were detected using an ECL western blotting kit.

### Transwell Assays and Wound Healing Assays

Cell migration and invasion were detected by Transwell methods. Cells (1–2 × 10^5^ cells) were suspended in FBS-free medium and then seeded into the top chamber (BD, United States). Medium with 10% FBS was added in the lower chamber. After incubation for 24–36 h, the chambers were fixed and stained with crystal violet. Evaluation of migration capacity was performed by counting invading cells under a microscope. The invasion assay was performed similarly to the migration assay with the upper chamber coated with Matrigel (BD Bioscience, United States).

Cells were cultured in 6-well plates until 90% confluence. Cell monolayer were scratched with pipette tip. Next, fresh serum-free medium was replaced. The scratch zones were photographed by inverted microscopy at different times. The data presented were repeated three times.

### Statistical Analysis

All statistical analyses were conducted with R (4.0.0) software and GraphPad Prism 8. All the results were presented as mean ± standard deviation (SD). The student’s t-test was performed to compare the differences between the two groups. Differences in survival between different risk groups were compared by Kaplan-Meier curves followed by a log-rank test. *p* < 0.05 was regarded as statistically significant.

## Results

### Data Source and Metastasis-Related lncRNAs

**FIGURE 2:**
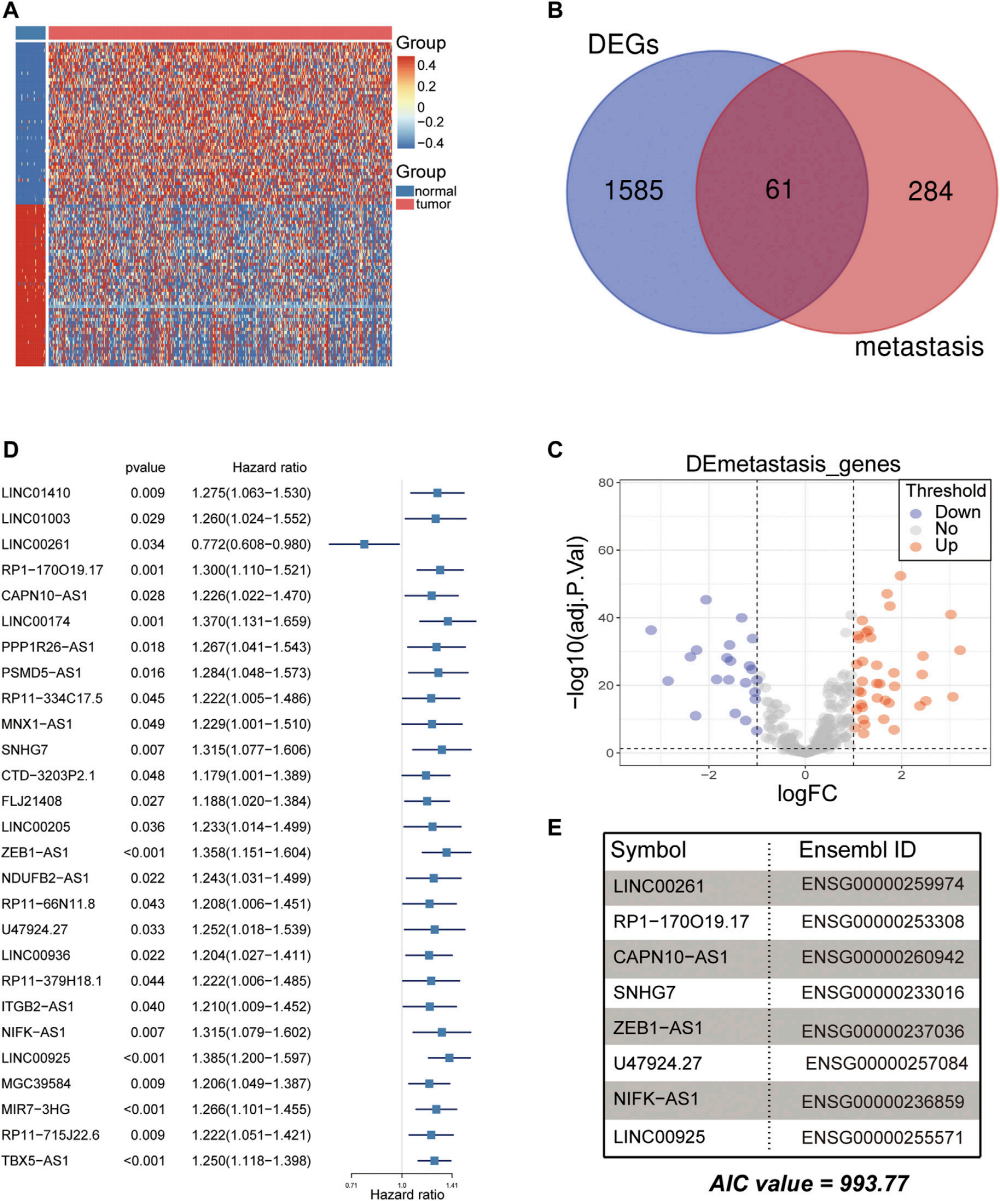
Analysis of differentially expressed metastasis-related genes for CRC. **(A)** Heatmap of the top 100 differentially expressed genes (DEGs) between normal and CRC tumor samples in the TCGA cohort. **(B,C)** The Venn plot and volcano plot showed that 61 metastasis-related genes were obtained in the TCGA cohort. Each red dot shows an upregulated gene, and each blue dot shows a downregulated gene (|Log2 Fold Change| > 1 and *p* < 0.05). **(D)** Twenty-seven lncRNAs were screened for prognostic significance using univariate Cox regression. **(E)** Eight metastasis-related lncRNAs were used to develop the signature using multivariate Cox regression (AIC value = 993.77).

The workflow of this study is shown in Figure 1. The gene expression and corresponding clinical data were downloaded from the TCGA and Gene Expression Omnibus (GEO) databases. Three eligible CRC cohorts, namely, TCGA (*N* = 448), GSE39582 (*N* = 585), and GSE29621 (*N* = 65), were integrated in our study for further analysis. The baseline information of all CRC datasets is summarized in Table 1, Supplementary Tables S2, S3. To determine the dysregulated genes in CRC tissues, we used RNA sequencing data from TCGA datasets. A heatmap of the top 100 differentially expressed genes (DEGs) is shown in Figure 2A. Then, a total of 61 potential prognostic metastasis-related genes were identified via the interaction network among these genes (Figures 2B,C). According to Pearson correlation analysis (correlation coefficient >0.4 and *p* < 0.05), we finally obtained 840 metastasis-related lncRNAs (Supplementary Table S1).

**TABLE 1:** The relationship between risk signature groups and clinical indexes in TCGA CRC cohort (*N* = 448).

TCGA	Total (*N* = 448)	High risk (*N* = 56)	Low risk (*N* = 392)	*p*
Age				0.856
≤65	187	24	163	
>65	261	32	229	
Gender				0.142
Female	209	21	188	
Male	239	35	204	
TNM stage				**0.007** **^a^**
Stage I-II	248	21	227	
Stage III-IV	189	32	157	
NA	11	3	8	

a Indicate statistically significant (*p* < 0.05). TCGA, The Cancer Genome Atlas; High risk, High risk group ; Low risk, Low risk group.

### Analysis of lncRNAs as Prognostic Biomarkers

**FIGURE 3:**
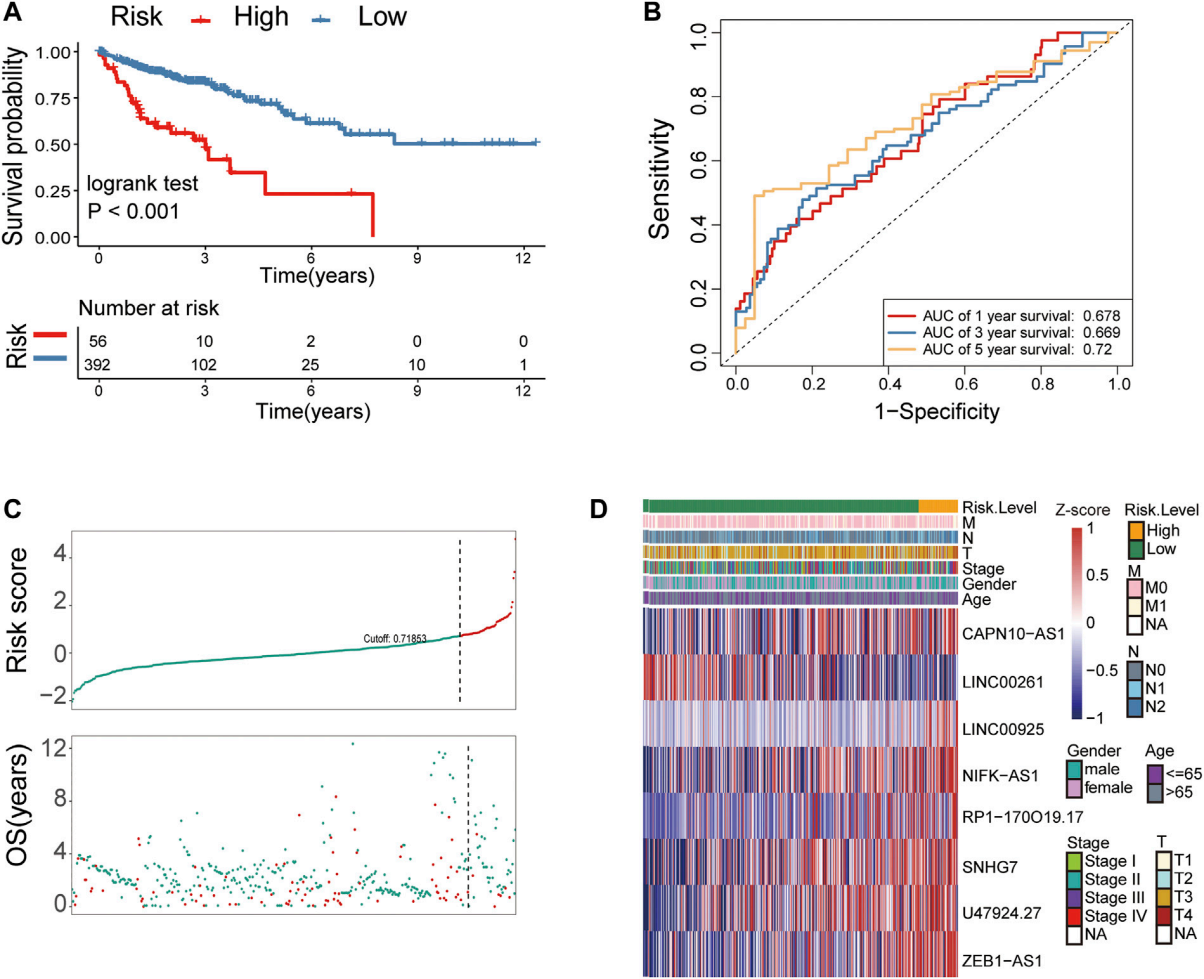
Identification of metastasis-related lncRNA prognostic signatures for CRC in the TCGA cohort. **(A)** Patients in the high-risk group (red) exhibited worse overall survival (OS) than those in the low-risk group (blue) in the TCGA cohort. **(B)** Receiver operator characteristic (ROC) curves predicted the sensitivity and specificity of 1-, 3-, and 5-year survival according to the 8-lncRNA signature-derived risk scores in the TCGA cohort. **(C)** The distribution and value of the risk scores in the TCGA cohort. **(D)** The expression of eight lncRNAs in CRC patients.

**FIGURE 4:**
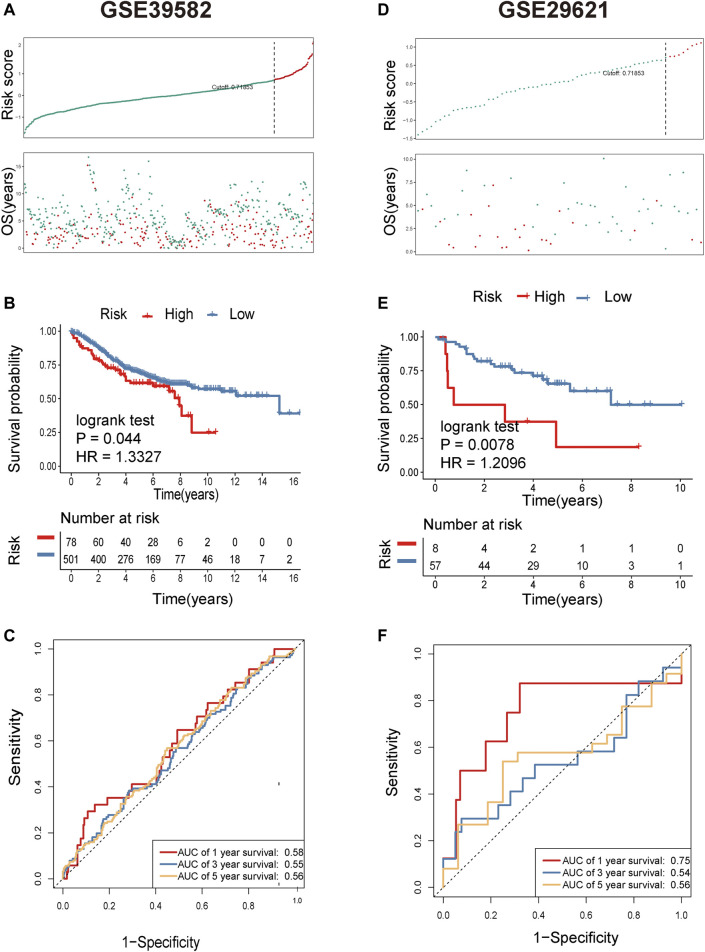
Identification of metastasis-related lncRNA prognostic signatures for CRC in the GEO cohort. **(A)** The distribution and value of the risk scores in the GSE39582 cohort. **(B)** Patients in the high-risk group (red) had worse overall survival (OS) than those in the low-risk group (blue) in the GSE39582 cohort. **(C)** Receiver operator characteristic (ROC) curves to predict the sensitivity and specificity of 1-, 3-, and 5-year survival according to the 8-lncRNA signature-derived risk scores in the GSE39582 cohort. **(D)** The distribution and value of the risk scores in the GSE29621 cohort. **(E)** Patients in the high-risk group (red) exhibited worse overall survival (OS) than those in the low-risk group (blue) in the GSE29621 cohort. **(F)** Receiver operator characteristic (ROC) curves to predict the sensitivity and specificity of 1-, 3-, and 5-year survival according to the 8-lncRNA signature-derived risk scores in the GSE29621 cohort.

Subsequently, the 840 metastasis-related lncRNAs were analysed via univariate Cox regression, and 27 lncRNAs were screened with prognostic significance (*p* < 0.05) **(** Figure 2D **)**. Based on multiple stepwise regression analyses, we finally obtained eight lncRNAs, namely, LINC00261, RP1-170O19.17, CAPN10-AS1, SNHG7, ZEB1-AS1, U47924.27, NIFK-AS1, and LINC00925 (Figure 2E; Table 2). These eight metastasis-related genes were also applied to construct a risk signature as follows: risk score = −0.2731 × exp(LINC00261) + 0.1864 × exp(RP1-170O19.17) −0.1764 × exp(CAPN10-AS1) −0.2143 × exp(SNHG7) + 0.3050 × exp(ZEB1-AS1) + 0.2794 × exp(U47924.27) + 0.2455 × exp(NIFK-AS1) + 0.3202 × exp(LINC00925). The cut-off value for the low-risk and high-risk groups was 0.7185, which was calculated by the R package survminer. We further explored the prognostic values of these genes, and the results suggested that patients with high RP1-170O19.17, CAPN10-AS1, SNHG7, ZEB1-AS1, U47924.27, NIFK-AS1, and LINC00925 expression had a significantly poorer prognosis (*p* < 0.05). In contrast, low LINC00261 expression predicted poor outcome in CRC (*p* < 0.05) (Supplementary Figures S1A–F). In addition, we investigated the expression of eight lncRNAs in CRC and normal tissues from the GEO database, and the results suggested that CAPN10-AS1, NIFK-AS1, SNHG7, and ZEB1-AS1 were highly expressed in CRC tissue compared with normal colon tissues, and Lnc00261 was significantly downregulated in CRC tissues (Supplementary Figures S2A–E, Table 3). Kaplan-Meier plots also showed that the mortality rate in the high-risk group was markedly higher than that in the low-risk group (Figure 3A). Then, the predictive value of the risk model was evaluated by ROC curves, and the AUCs for 1-, 3- and 5-year survival were 0.678, 0.669, and 0.72, respectively, (Figure 3B). The distributions of OS status are shown in Figure 3C. Heatmap analysis was used to visualize the expression of the eight lncRNAs in CRC patient samples (Figure 3D). To validate the robustness of the risk model, the validation cohort from GEO (GSE39582, GSE29621) was also stratified into high- and low-risk groups based on the same formula as that from the TCGA cohort. The results suggested that a higher risk score was also associated with poor OS (Figures 4A–F).

**TABLE 2:** Eight prognostic risk signatures closely related to metastasis.

LncRNA	Regression coefficient	Hazard Ratio (95%CI)	*p*
LINC00261	−0.273105584	0.7610 (0.5989–0.9670)	0.0254666
RP1-170O19.17	0.186355269	1.2049 (1.0341–1.4038)	0.0168732
CAPN10-AS1	−0.176378843	0.8383 (0.6585–1.0671)	0.1520407
SNHG7	0.21434717	1.2391 (0.9928–1.5464)	0.0579326
ZEB1-AS1	0.304989849	1.3566 (1.1020–1.6701)	0.0040356
U47924.27	0.279446187	1.3224 (1.0410–1.6799)	0.0220857
NIFK-AS1	0.245520969	1.2783 (1.0018–1.6312)	0.0483671
LINC00925	0.32023822	1.3775 (1.1823–1.6049)	4.02E-05

**TABLE 3:** The expression of eight lncRNAs in CRC and normal tissues from GEO database.

Gene	Analysis Id	(*n* (tumor *vs.* normal)	logFC	Average expression	*p*-value	Adjusted *p*-value
SNHG7	GSE83889	136 (101–35)	0.5617	5.7787	0	0
	GSE39582	585 (566–19)	0.4388	5.6487	0	0
	GSE21510	148 (123–25)	0.1979	6.1857	0	0
	GSE37364	65 (27–38)	0.299	6.662	0	0
	GSE50421	49 (24–25)	0.4339	8.4145	0	0
	GSE9348	82 (70–12)	0.3922	5.5273	0	0
	GSE31905	67 (60–7)	1.0363	7.9282	0.0002	0.001
	GSE22598	34 (17–17)	0.2325	6.3079	0.0002	0.0037
	GSE32323	34 (17–17)	0.2325	6.3079	0.0002	0.0037
	GSE81558	51 (42–9)	0.5831	8.4065	0.0002	0.0061
	GSE5206	105 (100–5)	0.2399	7.318	0.0038	0.0126
	GSE18105	111 (94–17)	0.1393	6.181	0.007	0.0143
	GSE71187	169 (157–12)	0.6637	5.6026	0.0048	0.0172
	GSE33113	96 (90–6)	0.2736	6.4665	0.0019	0.0192
ZEB1-AS1	GSE21510	148 (123–25)	0.869	5.3455	0	0
	GSE18105	111 (94–17)	0.8987	5.3567	0	0.0001
	GSE37364	65 (27–38)	0.5233	5.1887	0	0.0002
	GSE39582	585 (566–19)	0.5283	4.0832	0.0003	0.0029
	GSE83889	136 (101–35)	0.2574	4.8208	0.004	0.0177
	GSE9348	82 (70–12)	0.432	3.8851	0.0033	0.0236
CAPN10-AS1	GSE18105	111 (94–17)	0.2665	4.8794	0	0.0001
	GSE22598	34 (17–17)	0.2518	4.6532	0.0001	0.0025
	GSE32323	34 (17–17)	0.2518	4.6532	0.0001	0.0025
	GSE39582	585 (566–19)	0.2902	3.9924	0.0003	0.0034
NIFK-AS1	GSE21510	148 (123–25)	0.5147	6.1463	0	0
	GSE18105	111 (94–17)	0.5151	6.1071	0	0
LINC00261	GSE83889	136 (101–35)	−2.1856	6.5468	0	0
	GSE21815	141 (132–9)	−3.1328	8.9434	0	0
	GSE35279	79 (74–5)	−4.0417	8.9339	0	0.0001
	GSE50421	49 (24–25)	−1.1287	7.3291	0	0.0006
	GSE18105	111 (94–17)	−1.1704	7.7771	0.0003	0.0009
	GSE24713	68 (38–30)	0.5321	3.8319	0.0088	0.0214

### The Risk Score of the 8-lncRNA Signature is an Independent Prognostic Indicator

**FIGURE 5:**
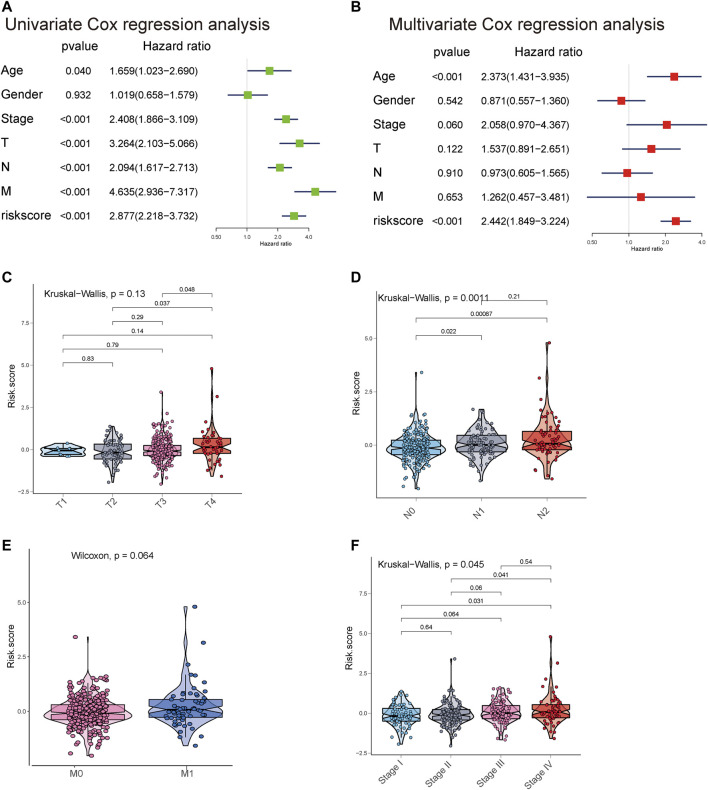
Univariate and multivariate analyses showed the prognostic value of the 8-lncRNA signature in the TCGA cohort. **(A,B)** Univariate and multivariate Cox regression analyses of the association between clinicopathological factors and OS of CRC patients. **(C–F)** The boxplot shows the risk score in T classification, N classification, M classification, and stage.

Univariate and multivariate Cox regression analyses were employed to determine whether the eight metastasis-related lncRNAs have prognostic value independent of clinicopathological indicators, such as age, pathological stage, and sex, in CRC. The results suggested that the risk score was significantly related to OS in the TCGA cohort (HR = 2.877, 95% CI = 2.218–3.732, *p* < 0.001). In multivariate Cox regression analyses, the risk score still proved to be an independent predictive factor for OS (HR = 2.442, 95% CI = 1.849–3.224, *p* < 0.001) (Figures 5A,B). In addition, we found that the risk score model was positively associated with N stage and M stage (Figures 5C–F). All these results suggested that this risk score model was an independent predictor for OS in CRC patients.

**FIGURE 6:**
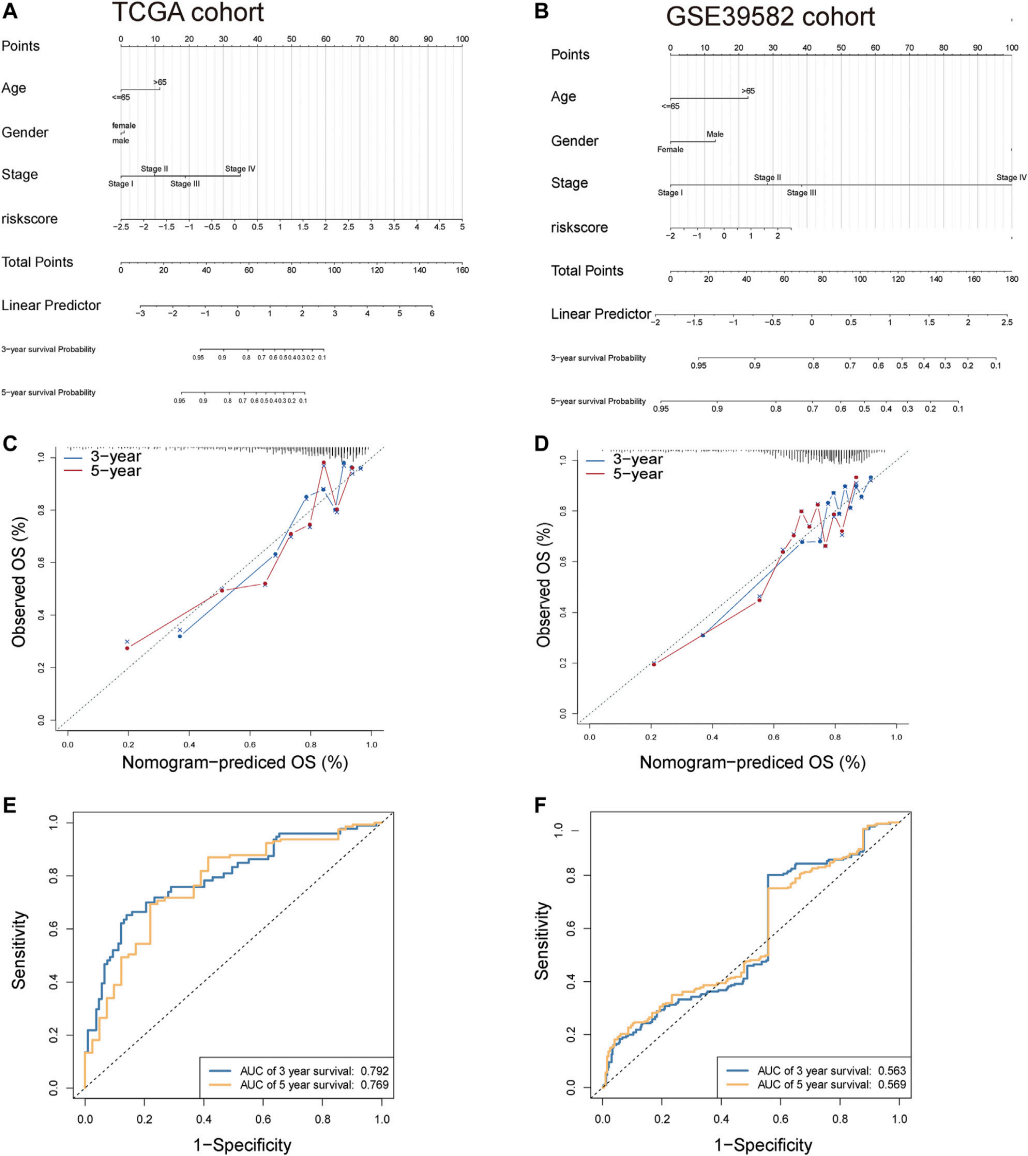
Construction of nomogram for OS at 3 and 5 years in CRC. **(A,B)** The nomogram consists of the clinical features (age, sex, and stage) and risk score. The variable scores were summed to give the total points, and the total point line is shown at the bottom of the nomogram. **(C,D)** Calibration plots were established to compare the proposed nomogram with an ideal model. **(E,F)** AUC experiments on the nomogram model suggested that this model had a higher accuracy in OS at 3 and 5 years.

Then, we performed the Fagan nomogram to examine the clinical application of the 8-lncRNA signature. All variables that were significant (age, sex, stage, and risk score) in the multivariate analysis are enumerated in the nomogram according to the algorithm. The variable scores were summed to give the total points, and the total point line is shown at the bottom of the nomogram, which can predict the probability of OS at 3 and 5 years (Figures 6A,B). Subsequently, calibration plots were established, which suggested that in comparison with an ideal model, the proposed nomogram had a similar performance (Figures 6C,D). Then, we performed AUC experiments on the nomogram model and found that this model had a higher accuracy in OS at 3-year survival (0.792, 0.769) and 5 years survival (0.563, 0.569) (Figures 6E,F).

### The 8-lncRNA Signature was Associated With Immune Cell Infiltration and the Mutational Landscape of CRC Patients

**FIGURE 7:**
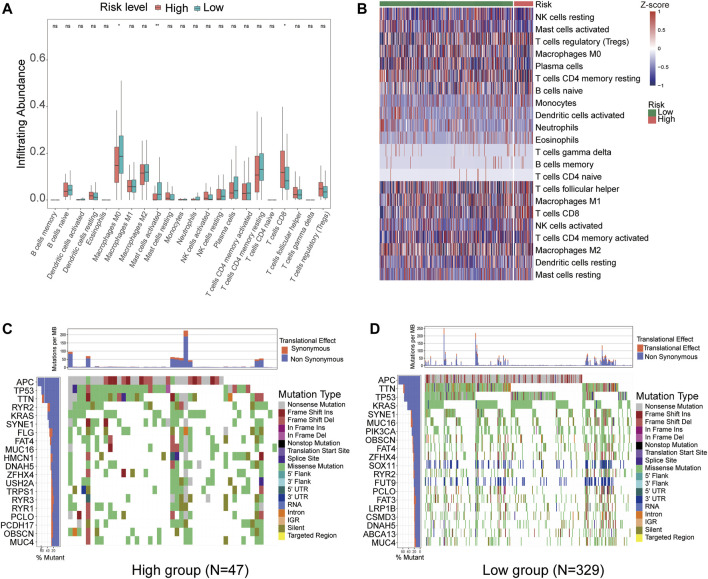
CIBERSORT identified the relative infiltration of 22 immune cell type subpopulations with different risk groups in the TCGA cohort. **(A)** The boxplot shows 22 immune cell types in different risk groups. **(B)** The relative infiltration of each cell type was normalized to the Z score. **(C,D)** The waterfall plot of tumor somatic mutations established by those with high-risk scores **(C)** and low-risk scores **(D)**.

To further explore the relationship between the prognostic model (risk score) and tumour-infiltrating immune-cell fractions, we quantified the enrichment scores of diverse immune cell subpopulations. The results demonstrated that compared to the high-risk group, patients in the low-risk group contained a higher proportion of memory CD8^+^ T activated and lower macrophage M0 and macrophage M2 activated (*p* < 0.05) (Figures 7A,B). We further investigated the specific mutational landscape of 376 CRC patients from the TCGA database. The waterfall plots were drawn by the Maftools package to illustrate the different mutated events of the two risk groups. The most detected genetic mutations were APC, TTN, KRAS, SYNE1, MUC16, OBSCN, FAT4, TP53, ZFHX4, RYR2, PCLO, and MUC4 mutations. Patients in the high-risk group presented higher mutation frequencies of RYR2, FLG, HMCN1, USH2A, TRPS1, RYR3, and RYR1 than those in the low-risk group (Figures 7C,D). These results suggest that the tumor mutation burden may be a new angle from which to provide an approach for specific immunotherapy.

### Eight-lncRNA Signature Can Predict the Response to Chemotherapy

**FIGURE 8:**
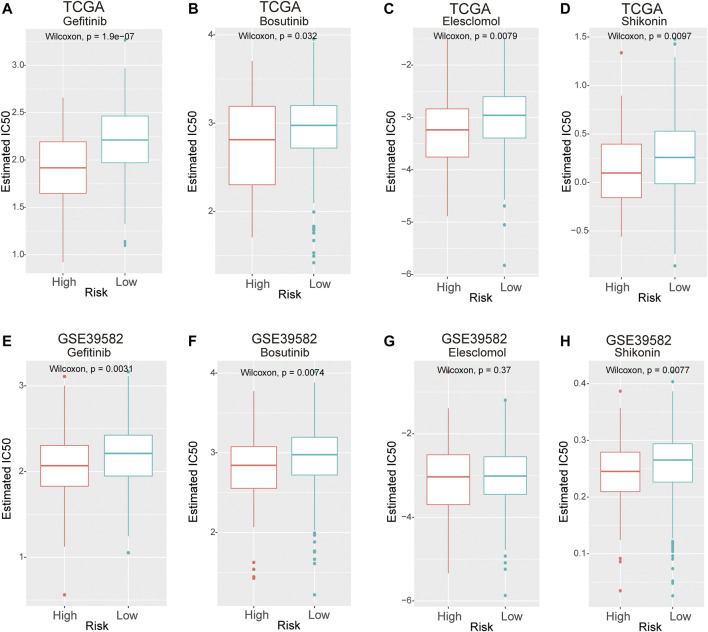
The IC50 values of four chemotherapeutic agents with an 8-lncRNA signature in the TCGA and GEO cohorts. **(A–D)** Gefitinib, camptothecin, bosutinib, and shikonin in the TCGA cohort. **(E–H)** Gefitinib, camptothecin, bosutinib, and shikonin in the GSE39582 cohort.

We used the R package “pRRophetic” to estimate the response to chemotherapy drugs in the high- and low-risk groups. Figure 8 shows the results of four commonly used chemotherapeutic agents for CRC. Our data showed that in contrast to patients in the low-risk group, the IC50 levels of gefitinib, bosutinib, elesclomol, and shikonin in the high-risk group were significantly lower than those in the low-risk group, which indicated that CRC patients in the high-risk group were more sensitive to these drugs (Figures 8A–D). To confirm the results, validation tests were conducted in the GSE39582 dataset, which suggested that patients in the high-risk group had lower IC50 values for gefitinib, bosutinib, and shikonin (Figures 8E–H).

### Functional Annotation Related to the Two Risk Groups

**FIGURE 9:**
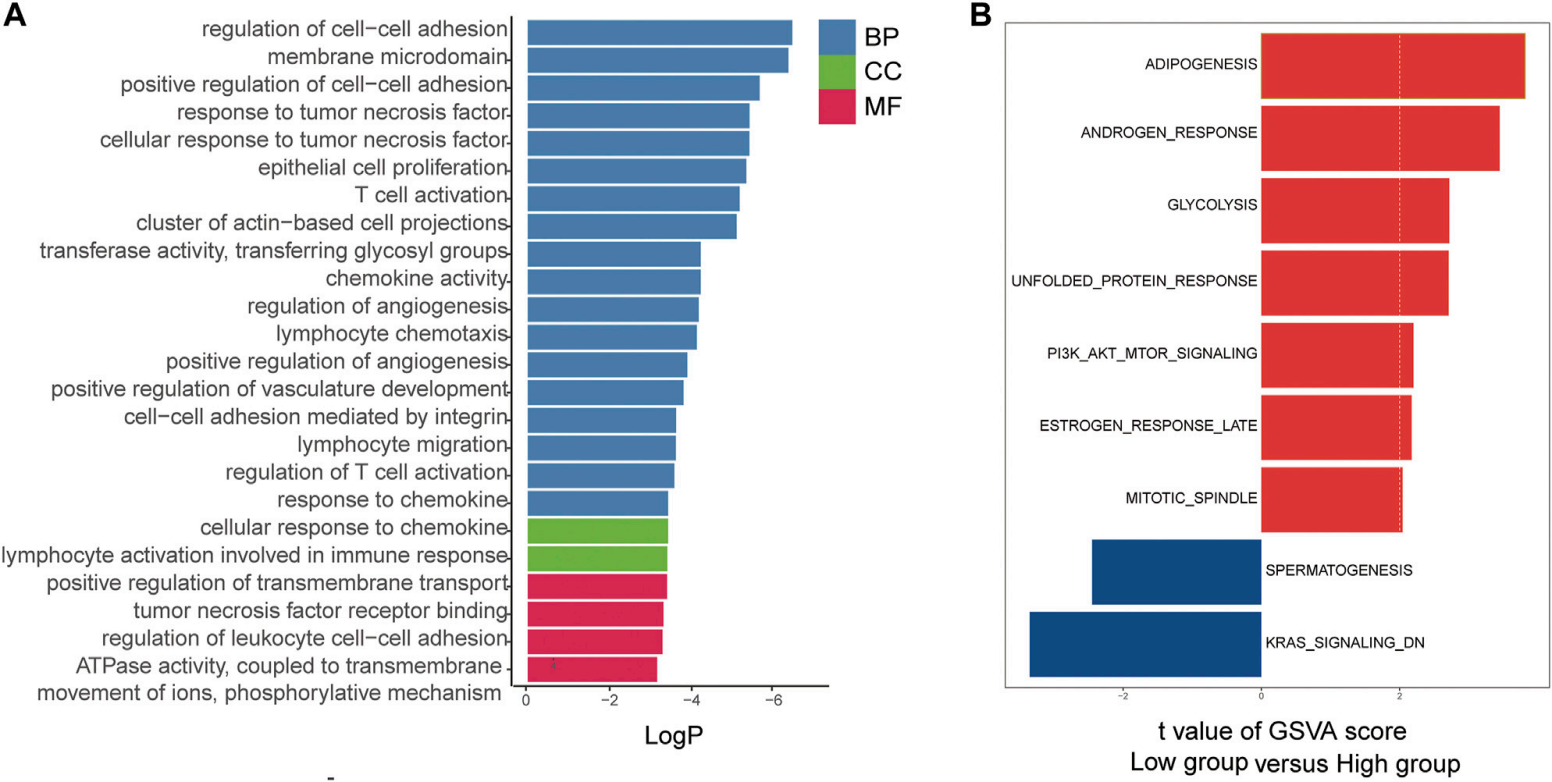
Functional annotation of the two risk groups in the TCGA cohort. **(A)** The GO enriched gene pathways/functions in distinct metastasis risk signature groups from TCGA CRC patients. **(B)** The hallmark enriched gene pathways/functions in distinct metastasis risk signature groups from TCGA CRC patients.

To investigate the potential biological functions and pathways associated with the risk signature, we performed GO and hallmark enrichment analyses between the high- and low-risk groups. Interestingly, as shown in Figures 9A,B, DEGs between the two groups were obviously enriched in a variety of metastasis-related pathways and functions, including cell−cell adhesion, membrane microdomain, cluster of actin-based cell projection, regulation of angiogenesis, cellular response to chemokines, positive regulation of transmembrane transport, lymphocyte migration, and cell and cell adhesion mediated by integrin. In addition, the DEGs were significantly enriched in the PI3K/AKT and KRAS signalling pathways.

### ZEB1-AS1 and SNHG7 Silencing Inhibited Colony Formation of CRC Cells *In Vitro*

**FIGURE 10:**
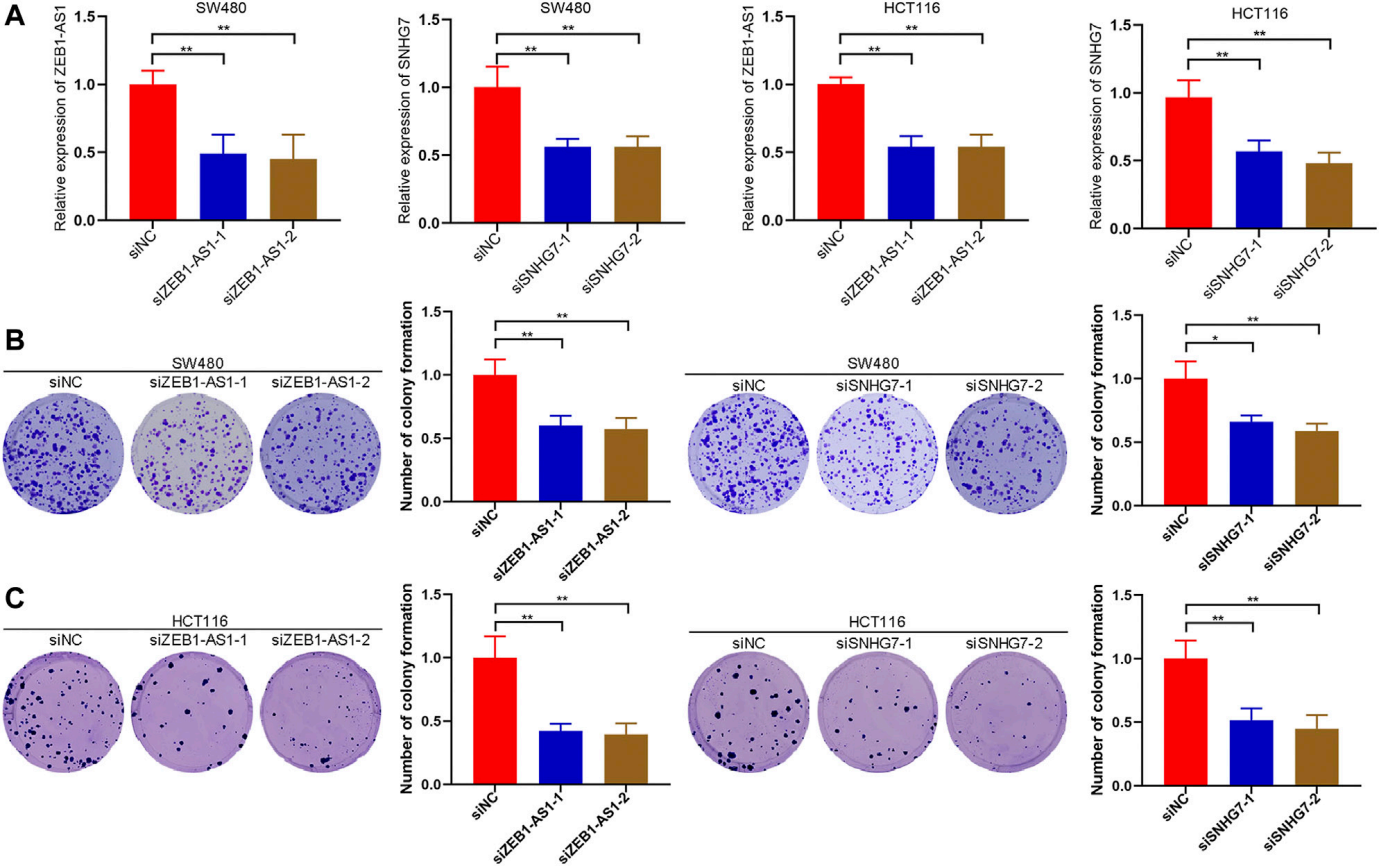
ZEB1-AS1 and SNHG7 silencing inhibited colony formation of CRC cells. **(A)** RNA interference (RNAi) was performed to deplete the expression of ZEB1-AS1 and SNHG7, and the transfection efficacy was then verified. **(B,C)** Colony formation assays suggested that downregulated expression of ZEB1-AS1 and SNHG7 could attenuate the colony formation of SW480 and HCT116 cells.

To validate the effect of lncRNAs on CRC cells, we next performed RNA interference (RNAi) to deplete the expression of ZEB1-AS1 and SNHG7, and the transfection efficacy was then verified (Figure 10A). A colony formation assay was performed to investigate the effect on cell growth, and the results suggested that downregulated expression of ZEB1-AS1 and SNHG7 could attenuate the colony formation of SW480 cells. Similar results were observed in ZEB1-AS1 and SNHG7-knockdown HCT116 cells (Figures 10B,C).

### ZEB1-AS1 and SNHG7 Silencing Inhibited the EMT Phenotype of CRC Cells

**FIGURE 11:**
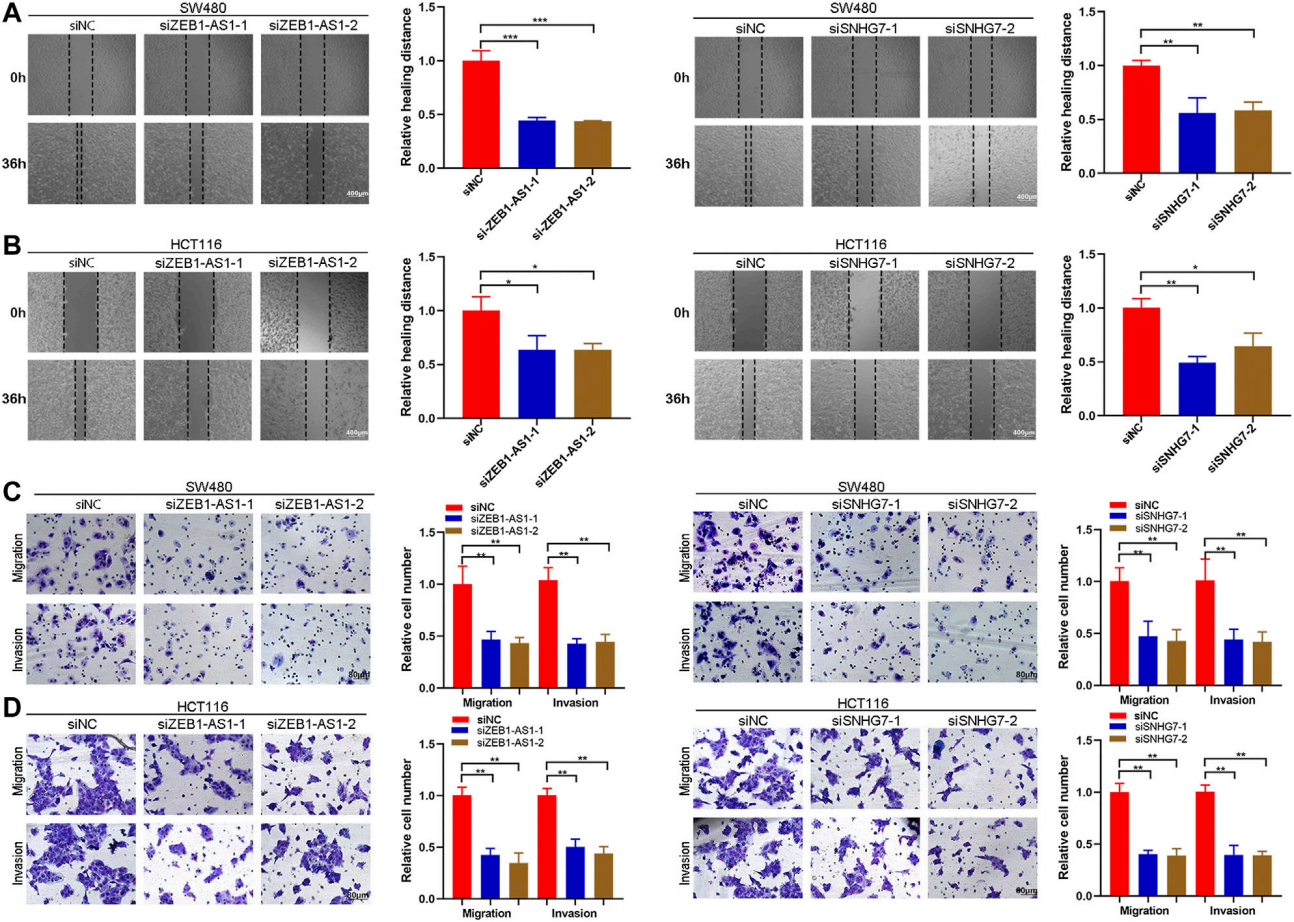
ZEB1-AS1 and SNHG7 silencing inhibited the migration and invasion of CRC cells. **(A,B)** Wound healing assay showing that cell migration was significantly reduced after ZEB1-AS1 and SNHG7-siRNA transfection. **(C,D)** Transwell migration and invasion assays were performed, and the results suggested that migration and invasion were significantly reduced in ZEB1-AS1 and SNHG7-siRNA-transfected SW480 and HCT116 cells.

**FIGURE 12:**
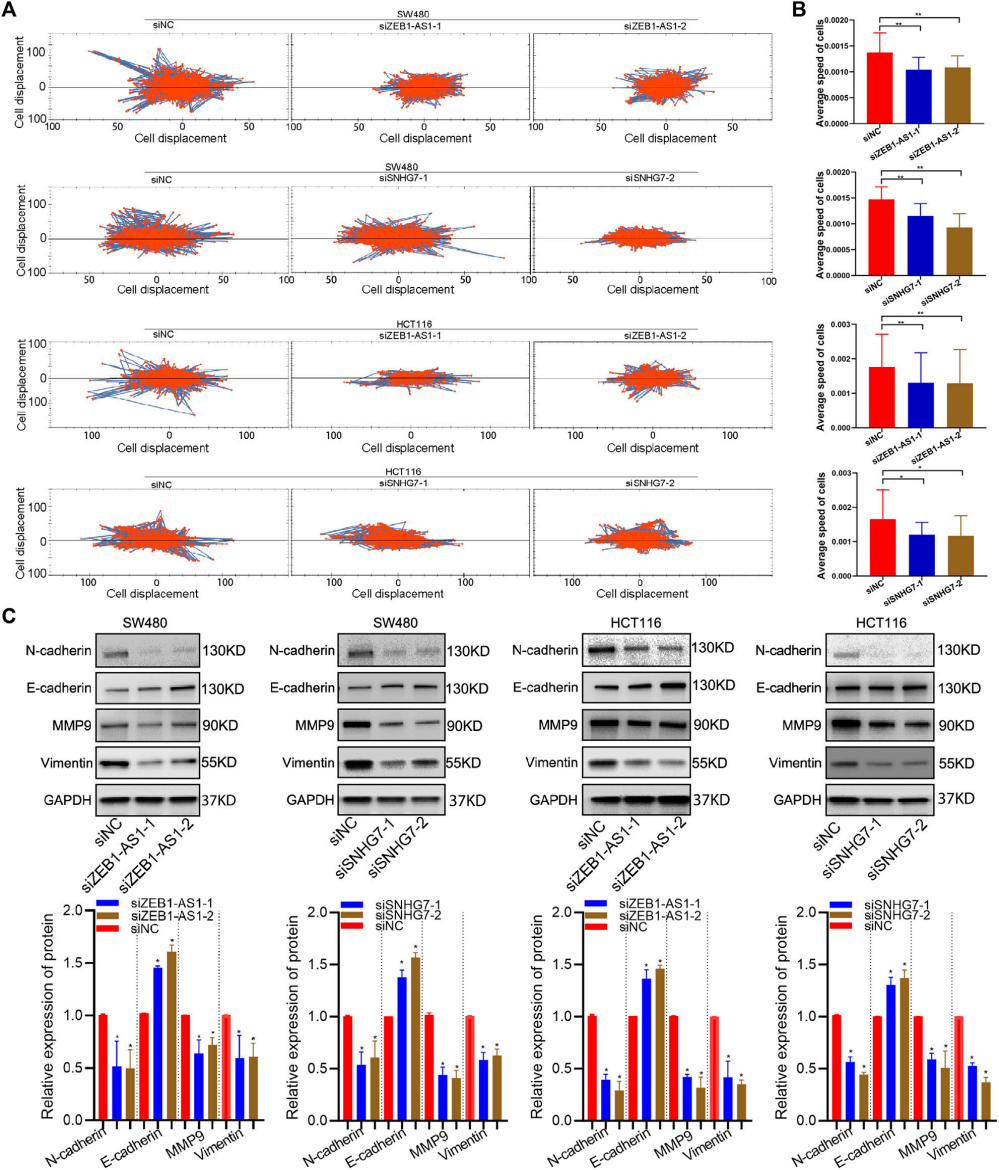
ZEB1-AS1 and SNHG7 silencing inhibited the EMT phenotype. **(A)** The High-Throughput Connotation of Imaging System was applied to continuously view the migration of cells, which suggested that ZEB1-AS1 and SNHG7-knockdown cells exhibited a lower cumulative displacement than control cells. **(B)** The transfected cells moved significantly slower on average than did the control cells. **(C)** ZEB1-AS1 and SNHG7 depletion increased the protein level of E-cadherin and decreased the expression of vimentin, N-cadherin, and MMP9.

Cell migration and invasion were evaluated by wound healing and Transwell assays. Wound healing analysis suggested that cell migration was significantly reduced after ZEB1-AS1 and SNHG7-siRNA transfection compared with the control group (Figures 11A,B). Transwell assays indicated that ZEB1-AS1 and SNHG7 knockdown suppressed migration and invasion in SW480 and HCT116 cells (Figures 11C,D). To further characterize the migratory behaviour, we applied the High-Throughput Connotation of Imaging System to continuously view the migration of cells. The results suggested that ZEB1-AS1 and SNHG7-knockdown cells exhibited a lower cumulative displacement than the control cells (Figure 12A). In addition, the transfected cells moved significantly slower on average than did the control cells (Figure 12B). These data suggested that these lncRNAs could impede cell growth migration and invasion in CRC cells. To further validate the effect of ZEB1-AS1 and SNHG7 on the EMT phenotype in CRC, we detected the expression of EMT-related genes in the transfected cells and found that ZEB1-AS1 and SNHG7 depletion increased the protein level of E-cadherin and decreased the expression of vimentin, N-cadherin, and MMP9 (Figure 12C). These data suggested that these lncRNAs could impede cell growth migration and invasion in CRC cells.

### ZEB1-AS1 and SNHG7 Modulated ERK/PI3K/AKT Pathways

**FIGURE 13:**
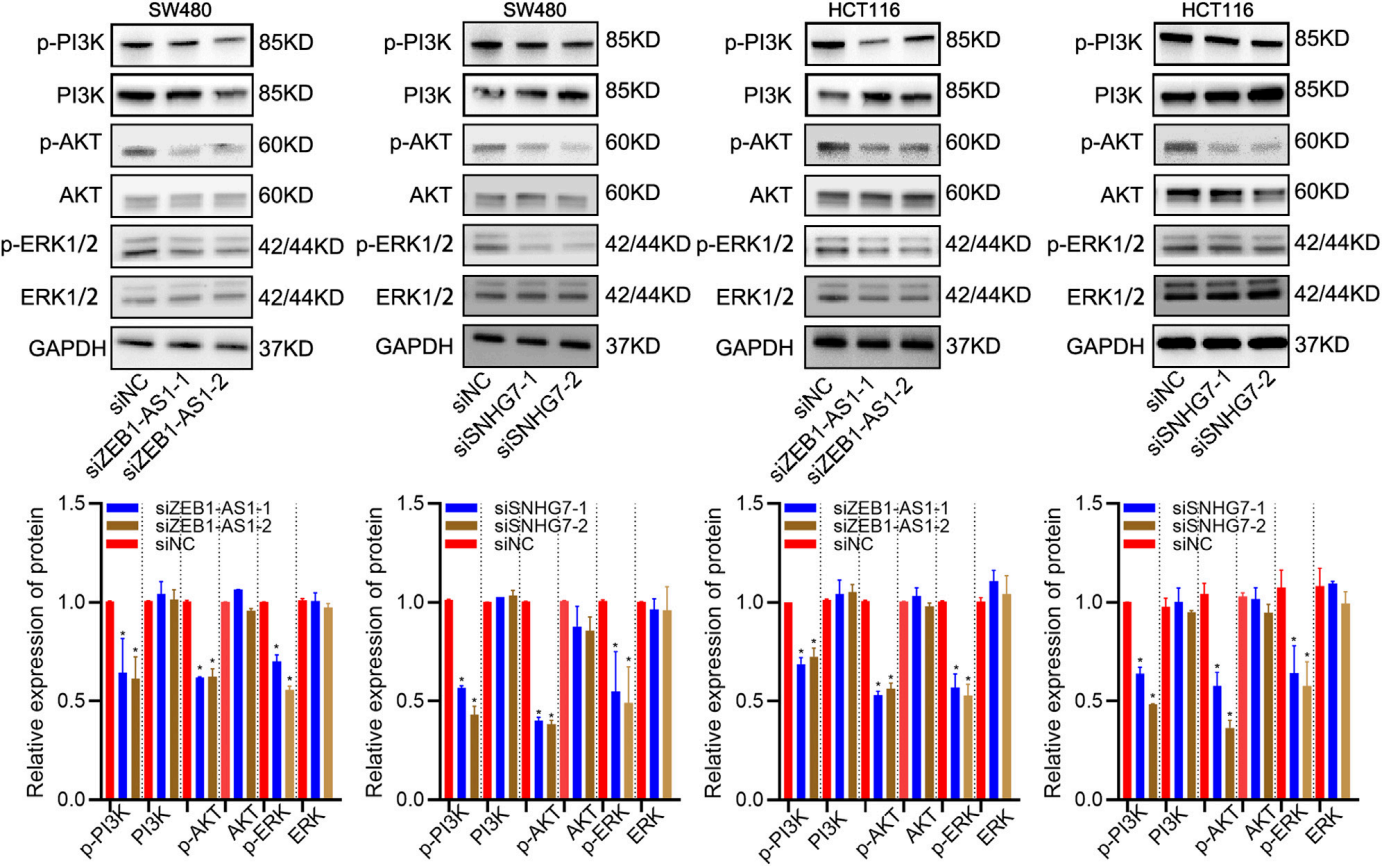
ZEB1-AS1 and SNHG7 modulated ERK/PI3K/AKT pathways. WB analysis suggested that ZEB1-AS1 and SNHG7 knockdown significantly decreased the levels of phospho-Akt (p-AKT), phospho-PI3K (P-I3K), and phospho-ERK (p-ERK).

In the enrichment analysis, we found that DEGs were significantly enriched in the PI3K/AKT pathways. This observation was further confirmed by WB analysis, which suggested that ZEB1-AS1 and SNHG7 knockdown significantly decreased the levels of phospho-Akt (p-AKT), phospho-PI3K (P-I3K) and phospho-ERK (p-ERK) (Figure 13). All these results suggested that ZEB1-AS1 and SNHG7 could modulate the EMT phenotype via ERK/PI3K/AKT pathways in CRC.

## Discussion

CRC is one of the most diagnosed malignancies and is considered a leading cause of cancer-related deaths worldwide (De Rosa et al., 2015; Siegel et al., 2017). Cancer metastasis and relapse may lead to increased chemoresistance and tumour progression, which may be the leading cause of death among patients with CRC (Guan, 2015). Therefore, it is urgent to explore potential mechanisms and reliable novel biomarkers for identifying and predicting the metastasis of CRC (Del Vecchio et al., 2017).

Undoubtedly, CRC patients with metastasis usually have a poor prognosis, with 5 years survival rates of less than 40% (Tsujimoto et al., 2019). Several classical biomarkers have been identified as metastasis-related signatures and exhibit high prognostic value in CRC patients (Zhang et al., 2020). Another report identified a seven key-gene signature (SPARC, COL1A2, MMP9, COL11A1, COL3A1, CXCL12, and THBS2) with a predictive logistic regression model, which may improve individualized outcome prediction in CRC (Tang et al., 2020). In addition, Wang and colleagues developed an EMT-related module composed of 51 gene pairs (51-GPS) to predict metastasis and relapse risk among patients with stage II CRC (Wang et al., 2020). In recent years, lncRNAs have been demonstrated to be involved in multiple biological processes of the metastatic cascade in CRC (Fenoglio et al., 2013). Several lncRNA models have been developed to predict the prognosis of CRC patients. However, few studies have investigated the metastasis-related lncRNA signature associated with the prognosis of CRC patients. In this study, we identified eight metastasis-associated lncRNAs and developed a metastasis-related signature for predicting the prognosis of CRC patients.

Owing to the clinical heterogeneity among CRC patients, single prognostic biomarkers could not be enough to make accurate predictions for the risk of metastasis. Integrating multiple biomarkers into a single prediction model may maximize the advantages of single biomarkers and the accuracy of prognostic prediction value across data sets. In the present study, eight lncRNAs, namely, LINC00261, SNHG7, ZEB1-AS1, NIFK-AS1, LINC00925, and RP1-170O19.17 CAPN10-AS1 and U47924.27 were found to be novel metastasis signatures by both univariate and multivariate Cox regression analyses, which also suggested that the expression of SNHG7, ZEB1-AS1, NIFK-AS1, LINC00925, and RP1-170O19.17 CAPN10-AS1 and U47924.27 were positively associated with the survival risk of CRC patients, while LINC00261 was negatively correlated with the survival risk. Then, we searched the related references and summarized the research status in tumorigenesis of the above eight genes included in the present prognostic signature as follows: LINC00261 has been investigated in many tumour types, including liver cancer, breast cancer, and CRC. Zhou et al. found that LINC00261 was significantly downregulated in CRC tissues and was clearly associated with clinical stage and lymph node metastasis (Zhou and Ma, 2019). Another study suggested that LINC00261 overexpression could reverse drug resistance and suppress cell migration, invasion, and proliferation (Wang et al., 2017). A recent study also demonstrated that overexpressed LINC00261 repressed CRC cell viability, migration, and invasion by downregulating nuclear *β*-catenin through inhibiting the activation of the Wnt pathway (Yan et al., 2019). Shintoku et al. Liu S also confirmed that LINC00261 was a metastasis-associated lncRNA that may be a novel candidate for the diagnosis and prognosis of CRC (Liu et al., 2020). Yi-Tian Chen found that NIFK-AS1 silencing could inhibit the proliferation, colony formation, and migration of hepatocellular carcinoma cells and sensitize tumor cells to sorafenib via upregulation of OATP1B1 and OATP1B3 (Chen et al., 2021). NIFK-AS1 was significantly decreased in tumour-associated macrophages of endometrial cancer patients (Zhou et al., 2018). Alessandra Santangelo identified NIFK-AS1 as a biomarker that could help screen potential groups that would benefit from regorafenib treatment (Santangelo et al., 2021). The expression of LINC00925 was markedly elevated in cervical cancer samples and extensively involved in cervical cancer development (Peng et al., 2016). SNHG7, a novel lncRNA, has been demonstrated to be an oncogene in a variety of human malignancies, including CRC (Chen and Shen, 2020). High expression of ZEB1-AS1 has been confirmed to be closely associated with poor prognosis in CRC patients. Liu et al. reported that SNHG7 knockdown decreased cell proliferation and induced cell apoptosis by inhibiting K-ras/ERK/cyclinD1 (Wang et al., 2017). ZEB1-AS1, an oncogenic regulator, has been demonstrated to exhibit a pivotal role in tumorigenesis and progression in hepatocellular carcinoma, oesophageal squamous cell carcinoma, glioma, osteosarcoma, bladder cancer, colorectal cancer, prostate cancer, and B-lymphoblastic leukaemia (Li J et al., 2018). Gong et al. reported that ZEB1-AS1 was highly expressed in CRC tissues and significantly correlated with the depth of tumor invasion and lymph node metastasis. Jingsun Wei identified ZEB1-AS1 as one of the eight autophagy-related lncRNA signatures, which could significantly predict the prognosis of CRC patients. Similarly, Bin Zhao constructed a risk score model to predict the prognosis of CRC patients based on two prognostic hub lncRNAs, MEG3, and ZEB1-AS1 (Zhao et al., 2020). However, these prediction modules were not experimentally validated. The specific mechanisms of these genes leading to tumorigenesis remain controversial.

In the present study, we investigated the expression of eight lncRNAs in CRC from the GEO database, and the results confirmed that CAPN10-AS1, NIFK-AS1, SNHG7, and ZEB1-AS1 were highly expressed in CRC tissue compared with normal colon tissues, and Lnc00261 was significantly downregulated in CRC tissues. Furthermore, we explored the prognostic values of these genes, which suggested that patients with high RP1-170O19.17, CAPN10-AS1, SNHG7, ZEB1-AS1, U47924.27, NIFK-AS1, and LINC00925 expression had a significantly poorer prognosis. In contrast, low LINC00261 expression predicted poor outcome in CRC. Moreover, a loss-of-function antisense approach was performed, and the results suggested that ZEB1-AS1 and SNHG7 silencing inhibited colony formation, migration, and invasion of CRC cells *in vitro*. WB analysis confirmed that this signature was significantly enriched in the PI3K/AKT pathways. Furthermore, this novel developed lncRNA signature could perfectly predict overall survival and was validated through two external study datasets. Therefore, these lncRNAs could be considered metastasis-related signatures predicting the prognosis of CRC.

A growing number of studies have highlighted the potential effects of the cancer immune microenvironment (CIM) on the development and progression of cancer. Assessment of the enrichment of tumour infiltrates may help to predict the prognosis and host immune response to tumour antigens of CRC patients (Töpfer et al., 2011; Maibach et al., 2020). In the present study, we found that compared to the high-risk group, the low-risk group had a higher proportion of activated memory CD8^+^ T cells and lower proportions of naïve (M0) macrophages and activated (M2) macrophages. In addition, we further performed GO analyses and unexpectedly found that several immune-related biological processes and pathways were enriched, which suggested that the 8-lncRNA metastasis signature may be a superior predictive determinant of tumour immune infiltration. CRC patients with tumour metastasis, especially distant metastasis, appear to be multidrug resistant to the current treatments (Fakih, 2015). Therefore, we assessed the effect of the 8-lncRNA signature model on the chemotherapeutic response. We found that patients in the high-risk group were insensitive to first-line chemotherapy drugs but sensitive to some nonconventional chemotherapeutic drugs, such as gefitinib, bosutinib, eleclomol, and shikonin, which explains why patients with metastasis tend to develop resistance to first-line treatment in CRC (Conlin and Seidman, 2008). Therefore, our gene signature would provide certain theoretical guidance for novel therapeutic approaches for CRC.

Nevertheless, there were several limitations in this study. Our study was conducted solely based on data from a public database, and the gene signature was identified mainly with retrospective datasets. Therefore, inherent case selection bias may have influenced the results. In addition, due to the limitation of the length of this paper, we only explored the expression of SNHG7 and ZEB1-AS1 and its biological functions in CRC, and the specific effect and mechanism of the other lncRNAs in CRC remain unclear. The expression profiles of the eight lncRNAs combined with clinical validation in the patients of the prospective cohort need to be further proven. These are relevant issues that will be explored further in follow-up research that will build on this initial study. More potential markers should be identified and incorporated into our prediction model in the future.

## Conclusion

In summary, we systematically analysed the expression profiles and prognostic value of the metastasis-related signature in CRC and developed an eight metastasis-related signature for predicting the prognosis of CRC patients. The eight metastasis-related signature was proven to be an independent prognostic factor for patients with CRC. Then, the lncRNA risk prediction score was validated through two external validation cohort. Nomograms were constructed, and the predictive accuracy was assessed by ROC curve analysis. This signature could effectively predict the immune status and chemotherapy response in CRC patients. Moreover, the potential roles of SNHG7 and ZEB1-AS1 as metastasis drivers in CRC were confirmed by *in vitro* experiments. Thus, the gene signature may serve as a novel biomarker with diagnostic and prognostic value for CRC patients.

## Data Availability

The datasets supporting the conclusions of this article are available in the Gene Expression Omnibus (GEO) database and the cancer genome atlas (TCGA) database.

## Supplementary Material

Supplementary Figure S1 Kaplan–Meier survival curve analysis for the metastasis-related lncRNAs **(A)** CAPN10-AS1, **(B)** LINC00261, **(C)** LINC00925, **(D)** NIFK-AS1, **(E)** RP1-170O19.17, **(F)** SNHG7, **(G)** U47924.27, and **(H)** ZEB1-AS1. The overall survival rate of identified metastasis-related lncRNAs was analysed using Kaplan–Meier survival curves.

Supplementary Figure S2 The expression of 8 lncRNAs in CRC and normal tissues from the GEO database suggested that CAPN10-AS1 **(A)**, SNHG7 **(B)**, NIFK-AS1 **(C)**, and ZEB1-AS1 **(D)** were highly expressed in CRC tissue compared with normal colon tissues, and Lnc00261 **(E)** was significantly downregulated in CRC tissues.
