# Supplementary figures and images for: Discovery and Validation of a Novel Metastasis-Related lncRNA Prognostic Signature for Colorectal Cancer
Source: Front Genet. 2022 May 19;13:704988. doi: 10.3389/fgene.2022.704988 (PMC9162157; doi:10.3389/fgene.2022.704988)

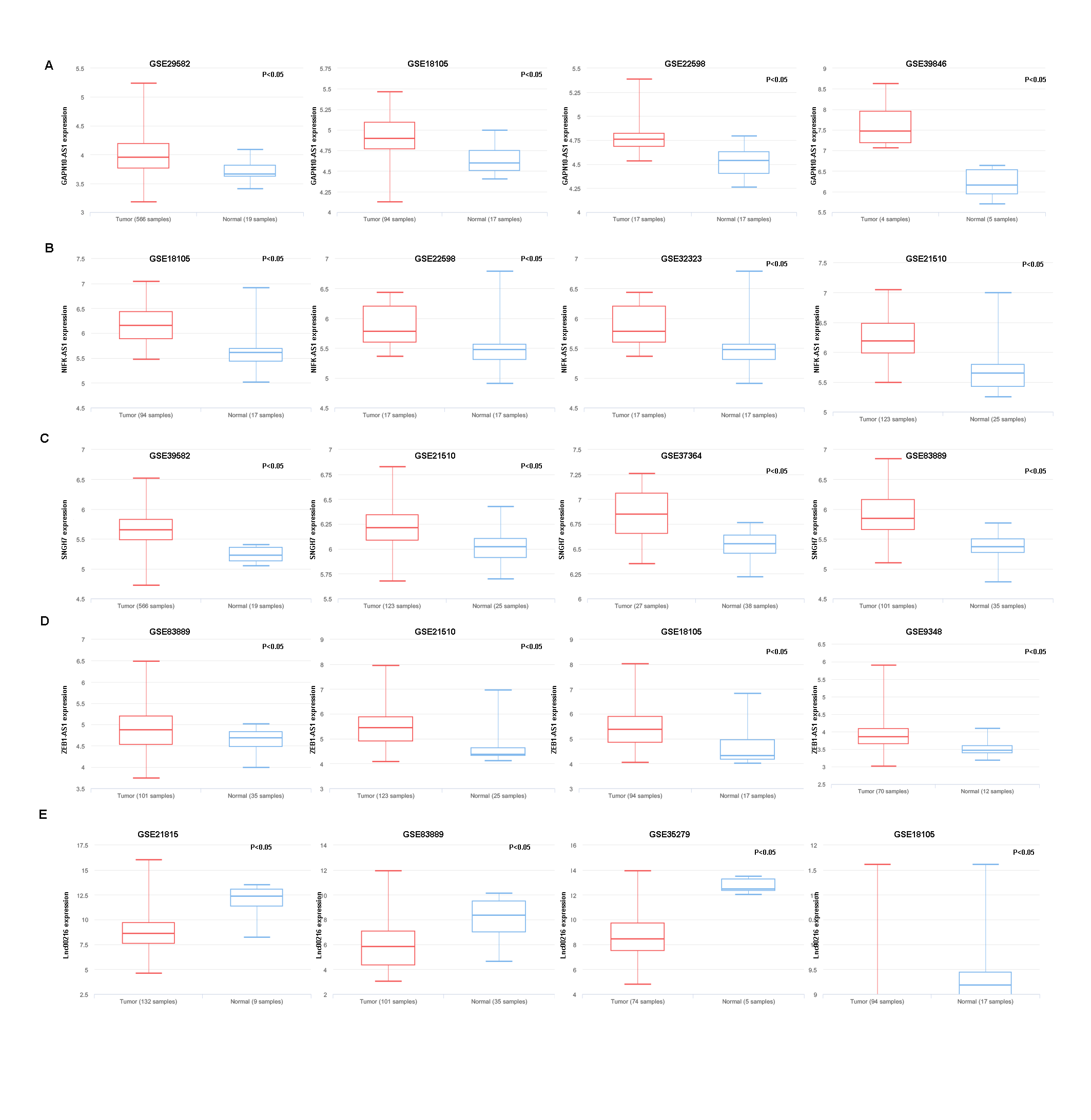

Supplement: Supplementary file 1 [file Image2.TIF]

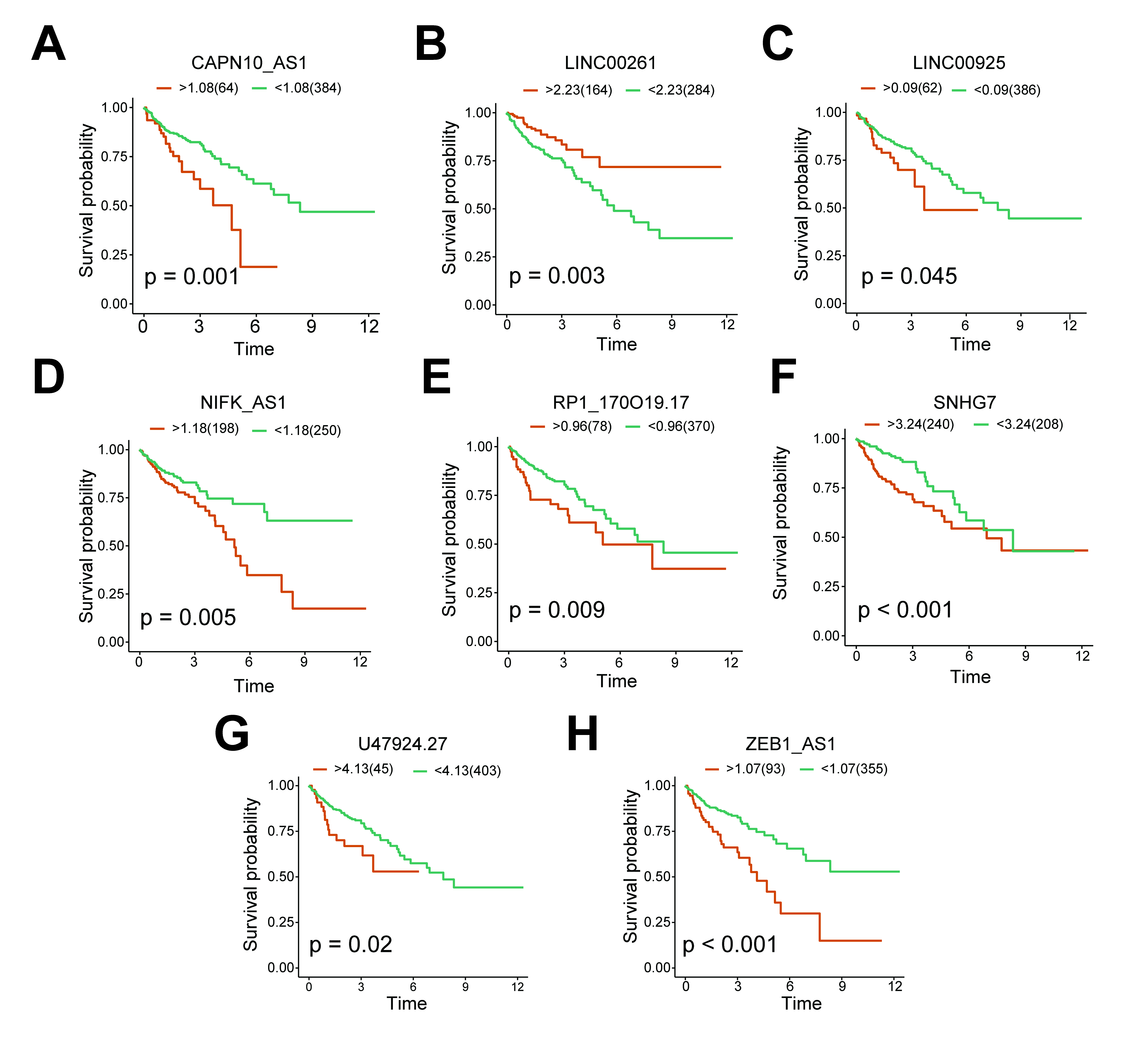

Supplement: Supplementary file 2 [file Image1.TIF]
