# Supplementary material for: Discovery and Validation of a Novel Metastasis-Related lncRNA Prognostic Signature for Colorectal Cancer
Source: Front Genet. 2022 May 19;13:704988. doi: 10.3389/fgene.2022.704988 (PMC9162157; doi:10.3389/fgene.2022.704988)
Supplement: Supplementary file 3 [file DataSheet2.docx]

**Table S2**. The relationship between risk signature groups and clinical indexes in GEO (GSE39582) CRC cohort (*N* = 585).

| GEO | Total (*N*=585) | High risk (*N*=78) | Low risk (*N*=507) | P |
| --- | --- | --- | --- | --- |
| Age |  |  |  | 0.717 |
| ≤65 | 228 | 29 | 199 |  |
| ＞65 | 356 | 49 | 307 |  |
| NA | 1 | 0 | 1 |  |
| Gender |  |  |  | 0.473 |
| Female | 263 | 38 | 225 |  |
| Male | 322 | 40 | 282 |  |
| TNM stage |  |  |  | 0.135 |
| Stage Ⅰ-Ⅱ | 309 | 35 | 274 |  |
| Stage Ⅲ-Ⅳ | 270 | 42 | 228 |  |
| NA | 6 | 1 | 5 |  |

**Table S3**. The relationship between risk signature groups and clinical indexes in GEO (GSE29621) CRC cohort (*N* = 65).

| GEO | Total  (*N*=65) | High risk (*N*=8) | Low risk (*N*=57) | P |
| --- | --- | --- | --- | --- |
| Gender |  |  |  | 0.471 |
| Female | 25 | 2 | 23 |  |
| Male | 40 | 6 | 34 |  |
| TNM stage |  |  |  | 0.066 |
| Stage Ⅰ-Ⅱ | 29 | 1 | 28 |  |
| Stage Ⅲ-Ⅳ | 36 | 7 | 29 |  |
